# Targeting Receptor-Type Protein Tyrosine Phosphatases with Biotherapeutics: Is Outside-in Better than Inside-Out?

**DOI:** 10.3390/molecules23030569

**Published:** 2018-03-02

**Authors:** Yotis A. Senis, Alastair J. Barr

**Affiliations:** 1 Institute of Cardiovascular Sciences, College of Medical and Dental Sciences, University of Birmingham, Birmingham B15 2TT, UK; y.senis@bham.ac.uk; 2 Department of Biomedical Science, Faculty of Science and Technology, University of Westminster, London W1W 6UW, UK

**Keywords:** tyrosine phosphatase, receptor, phosphorylation, ectodomain, biotherapeutic, biological, inhibitor, disease

## Abstract

Protein tyrosine phosphatases (PTPs), of the receptor and non-receptor classes, are key signaling molecules that play critical roles in cellular regulation underlying diverse physiological events. Aberrant signaling as a result of genetic mutation or altered expression levels has been associated with several diseases and treatment via pharmacological intervention at the level of PTPs has been widely explored; however, the challenges associated with development of small molecule phosphatase inhibitors targeting the intracellular phosphatase domain (the “inside-out” approach) have been well documented and as yet there are no clinically approved drugs targeting these enzymes. The alternative approach of targeting receptor PTPs with biotherapeutic agents (such as monoclonal antibodies or engineered fusion proteins; the “outside-in” approach) that interact with the extracellular ectodomain offers many advantages, and there have been a number of exciting recent developments in this field. Here we provide a brief overview of the receptor PTP family and an update on the emerging area of receptor PTP-targeted biotherapeutics for CD148, vascular endothelial-protein tyrosine phosphatase (VE-PTP), receptor-type PTPs σ, γ, ζ (RPTPσ, RPTPγ, RPTPζ) and CD45, and discussion of future potential in this area.

## 1. Introduction

**Figure 1:**
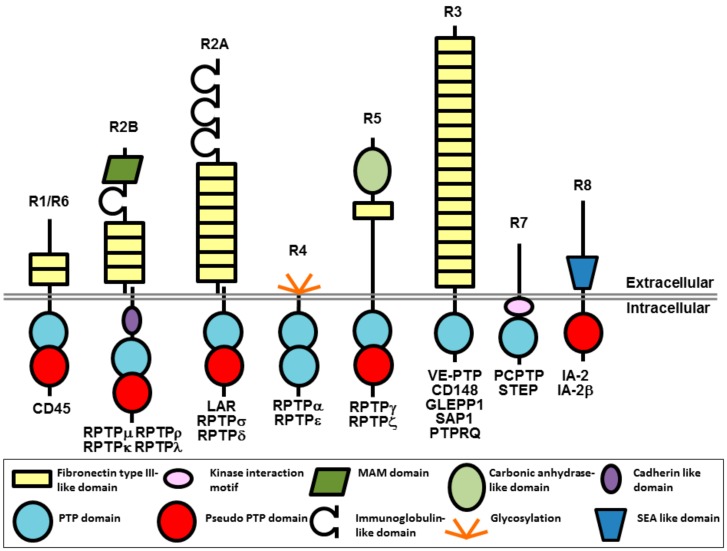
Schematic representation of RPTPs (adapted by permission from N.K. Tonks, Nature Reviews Molecular Cell Biology; published by Springer Nature, 2006, reference [3]). The gap at the extracellular juxtamembrane region of R2B and R2A represents the potential of these RPTPs to be proteolytically cleaved into two subunits that remain non-covalently associated at the cell surface [4]. MAM, Meprin/A5/μ domain; SEA, sea urchin sperm protein/enterokinase/agrin.

Protein tyrosine phosphorylation is a key signaling event that regulates cellular pathways involved in a wide range of physiological processes in the cardiovascular, immune, neuronal, and metabolic systems of the body. The overall level of tyrosine phosphorylation is determined by the balanced actions of kinases and phosphatases. Of the 37 classical human protein tyrosine phosphatases (PTPs), 21 are receptor-like transmembrane proteins and 16 are intracellular non-transmembrane proteins [1,2]. Receptor-type protein tyrosine phosphatases (RPTPs), which are the focus of this review, are grouped into 8 subtypes (R1–R8) and have a single transmembrane spanning domain, variable N-terminal extracellular regions and either a single or tandem intracellular phosphatase domain (Figure 1). The diverse extracellular regions have a modular architecture composed of multiple domains, often found in cell-adhesion molecules that enable extracellular events to be conveyed to intracellular signaling. On the intracellular side, most RPTPs have a tandem arrangement of PTP domains with the membrane proximal domain possessing full catalytic activity, while the distal domain has either weak activity, or is catalytically inactive.

Currently we have a detailed understanding of the structure and mechanism of phosphatase domains; however, our knowledge of the structure of RPTP ectodomains and understanding of their role in regulating function is incomplete. Many mechanisms for the regulation of RPTPs have been proposed, including activatory and inhibitory ligands, dimerization-induced inactivation, and size-exclusion models (these and other mechanisms have been reviewed elsewhere [3,5,6]). There is no unifying model of regulation and while some RPTPs have multiple ligands, many others are orphan receptors, or are thought to function in the absence of a ligand. To inform discussion in later sections of this article, we provide a brief explanation of the inhibitory wedge model and the head-to-toe dimerization model. Both models propose RPTP regulatory mechanisms involving dimerization and inhibition. The inhibitory wedge model was initially proposed based on the crystal structure of a single PTP catalytic domain from PTPRα, which crystallized as a dimer with a sequence near the N-terminus, the wedge, occluding the active site of the other PTP domain in the dimer [7,8]. Subsequent experiments with other full-length RPTPs demonstrated that homodimerization led to inhibition of phosphatase activity leading to the hypothesis that this is a general regulatory mechanism involving the wedge region. The secondary structure of this region (helix-turn-helix) is conserved in all classical PTPs although amino acid conservation is low [2]. However, this model is controversial since it is incompatible with structures of tandem PTP domains from multiple RPTPs [9,10], and the inhibited dimeric state involving the wedge has not been observed in any other PTP structure [10]. An alternative and distinct model of regulation for RPTPγ and RPTPζ proposes head-to-toe homo-dimerization of the tandem phosphatase domain in which the active site of the active phosphatase domain is occluded by the distal phosphatase domain from the dimer partner, leading to inhibition of phosphatase activity [10].

Research over the past decades has established that aberrant PTP signaling, through genetic mutation or altered expression levels, is associated with many human diseases and PTP knock-out mice studies have identified potential therapeutic opportunities for PTP inhibitors (reviewed in [11,12,13,14,15]). The challenges associated with small molecule PTP drug development have been well documented and as yet there are no clinically approved drugs targeting PTPs, although recent notable developments have included the identification of the compound SHP099 [16], a src homology 2-containing protein-tyrosine-phosphatase (SHP2) inhibitor for treatment of cancers, and low-molecular-weight PTP (LMPTP) inhibitors for diabetes [17]. Here we provide an update on the emerging area of receptor PTP-targeted biotherapeutics for CD148, VE-PTP, RPTPσ, CD45, RPTPγ, and RPTPζ, and discussion of future potential in this area.

## 2. Background on Currently Used Biotherapeutics

Therapeutic antibodies offer many advantages as is evident from the rapidly increasing number of antibodies in clinical use and the significant number in development [18]. For RPTPs, antibodies may be directed at less well conserved regions of the ectodomain with high specificity and affinity, avoiding off-target toxicity. Monoclonal antibodies, and other biotherapeutics, can have various modes of action that may not be achieved with small molecules [19]. For example, binding of the antibody to a ligand, either soluble or cell-associated, can interfere with activation of the therapeutic target. Bevacizumab (anti-vascular endothelial growth factor, VEGF), infliximab (anti-tumor necrosis factor, TNF), and eculizumab (anti-complement C5) are examples of approved antibodies with this mechanism of therapeutic action in oncology, inflammatory disease and haematology, respectively. In addition, antibodies may stabilize a distinct inactive receptor conformation, promote internalization of a cell-surface receptor, block ligand binding by directly competing for the ligand binding site, or modulate the oligomerization state of a receptor. Examples of such antibodies include Trastuzumab (human epidermal growth factor receptor, HER2-blocking antibody) and cetuximab (epidermal growth factor receptor, EGFR-blocking). Other functional effects of antibodies may be mediated via the crystallisable fragment (Fc) region mediating cell killing functions. In addition monoclonal antibodies often have a much longer half-life than small-molecule compounds, allowing reduced dosing frequency [20]. Variation in plasma concentration among individuals can be lower in comparison to small molecules [21]. During the drug development phase, it has been documented that the rate of attrition is much lower than for small molecules [22]; however, they do have the limitation that manufacturing costs are significantly higher.

## 3. Therapeutic Potential of Biotherapeutics Targeting CD148 (*PTPRJ*)

CD148, initially referred to as density enhanced phosphatase-1 or DEP-1 [23], is a member of the R3 subtype of receptor PTPs together with vascular endothelial-protein tyrosine phosphatase (VE-PTP), glomerular epithelial protein 1 (GLEPP1), stomach-cancer-associated protein tyrosine phosphatase 1 (SAP1), and receptor-type tyrosine-protein phosphatase Q (PTPRQ). Structurally this group of RPTPs is defined by an ectodomain composed of multiple fibronectin type III-like repeats and a single catalytic phosphatase domain (Figure 1). The number of FNIII-like repeats ranges from 9 in CD148 to 17 in VE-PTP. CD148 is broadly expressed in both haematopoietic cells (platelets, T cells, B cells, and macrophages) and non-haematopoietic cells (endothelial cells, fibroblasts and smooth muscle cells, and thyroid cells).

**Figure 2:**
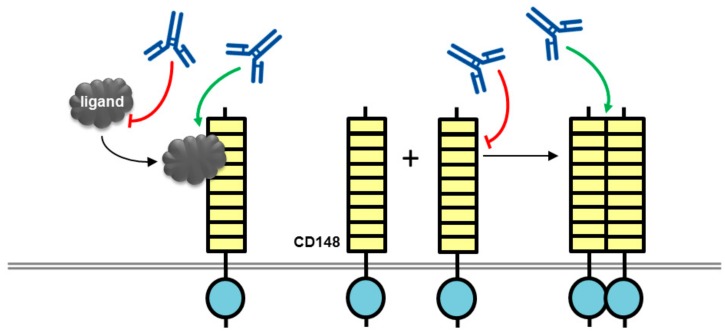
Potential mechanisms of action for CD148 antibodies with biological effects. An antibody (blue lined object) directed to the ligand binding domain, the ligand itself or the ligand-receptor complex may modulate ligand-mediated increases in phosphatase activity. Alternatively, antibodies may modulate phosphatase activity by inducing or inhibiting oligomerization, which is thought to regulate catalytic activity. RPTP domains are represented by objects as detailed in Figure 1.

Takahashi et al. developed a monoclonal antibody (Ab1) against the ectodomain of CD148 and investigated the effect of this antibody on endothelial cell growth [24]. The antibody suppressed serum-stimulated growth of endothelial cell lines and blocked vessel formation in an in vivo mouse corneal angiogenesis assay. It is noteworthy that these effects were observed only with the bivalent (intact) antibody and not with the monovalent (Fab fragment). Investigation of the antibody’s mechanism of action in biotin surface labelling experiments ruled out effects on CD148 cell surface expression, while experiments in Chinese hamster ovary (CHO) cells expressing CD148 constructs revealed that the antibody’s biological effect was absent in cells expressing a catalytically inactive CD148 (a construct in which the key catalytic cysteine residue of the active site is mutated to serine; C/S mutant), or a CD148 construct lacking the cytoplasmic domain, indicating that a functional phosphatase domain is required for the biological effect. Analysis of the effect of the antibody on the phosphorylation status of CD148 substrates (ERK1/2 and c-Met) indicated that the antibody increased CD148-associated phosphatase activity leading to a decrease in substrate phosphorylation. Again, this effect was observed only with bivalent antibody and not Fab fragment, and effects on other phosphoproteins that are not CD148 substrates were absent. An antibody-dependent block of cell cycle progression (G0/G1 phase) was also reported, which correlates with the inhibition of cell proliferation. The authors suggested that ectodomain oligomerization induced by the bivalent antibody, but not with the Fab fragment, led to increased phosphatase activity and this was the potential mechanism of the biological effects of the antibody (Figure 2). Although a modest increase in phosphatase activity was observed following incubation of cells with the bivalent antibody, an effect on the oligomerization state of CD148 was not reported and there are potentially several other mechanisms that may be involved, as we discuss below.

One potential mechanism of action for this antibody may have involved modulation of ligand binding to the ectodomain, although further investigation is required to determine the mechanistic basis of this effect. Two CD148 ligands have now been reported, thrombospondin-1 (TSP-1) and syndecan-2, although these were not known at the time of publication of the studies on the Ab1 antibody [25,26]. Both ligands have similar effects on cell growth: TSP-1 mediates increased CD148 catalytic activity, leading to dephosphorylation of substrate proteins, and inhibition of endothelial cell growth; while interaction of CD148 with syndecan-2 stimulates cell adhesion and focal adhesion formation potentially leading to down-regulation of cell proliferation and growth. One can speculate that an effect of the Ab1 antibody on increasing the magnitude or duration of ligand effects would be consistent with its biological actions (Figure 2).

Although Ab1 antibody-induced changes in CD148 cell surface expression were negligible, it is possible that modulation of CD148 activity by the antibody may have led to changes in the cell surface expression and activity of growth factor receptors that interact with CD148, thereby leading to the biological effect. Tarcic et al. and other groups, have demonstrated that CD148 suppresses signals from various growth factors receptors (platelet-derived growth factor receptor; hepatocyte growth factor receptor, vascular endothelial growth factor receptor (VEGFR), and epidermal growth factor receptor (EGFR), and in the case of the EGF receptor, CD148 inhibits phosphorylation-dependent activation of the receptor at the plasma membrane and translocation of the receptor to endosomes. It is the latter event, translocation of the active receptor, that supports long-term signaling [27]. Silencing of CD148 with siRNA leads to a loss of the physical interaction of EGFR and CD148, causing enhanced EGF responses, such as activation of the mitogen activated protein kinase cascade, and promotion of cell proliferation. Similarly, a CD148 antibody that disrupts the interaction of a growth factor receptor tyrosine kinase with CD148 might be expected to enhance growth factor responses. How this might correlate with the effects of the Ab1 antibody, namely increased phosphatase activity and an inhibition of cell proliferation, are unclear. One possible explanation may be linked to the report by Brunner et al. that CD148 down-regulates the urokinase receptor (uPAR), which is thought to be essential for endothelial cell proliferation and angiogenesis [28].

The Ab1 antibody epitope on the CD148 ectodomain is of particular interest with regard to potentially explaining the mechanistic basis of the antibody action. The epitope was mapped to an 8 amino acid sequence (324-QSRDTEVL-331) which lies within what is predicted to form the 3rd fibronectin III-like domain of CD148 (amino acids 271-364) [24]. The region has two single nucleotide polymorphisms that give rise to nonsynonymous substitutions: rs1566734 (A1176C, Q276P) and rs1503185 (G1326A, R326Q). These polymorphisms show a strong linkage disequilibrium and it has been suggested that the presence of 276P and 326Q might be associated with lower phosphatase activity. In one study of *PTPRJ* alleles and colorectal cancers a significant loss of the A allele (A1176C, Q276) was detected, suggesting that this may be a cancer resistance allele based on the suggestion that CD148 has tumour suppressor functions, and that the C allele (276P form of CD148) has reduced activity [29]. Similarly, Rollin et al. concluded that platelets from patients with the 276P/326Q alleles of CD148 were hypo-responsive to activating stimuli, associated with reduced CD148 activity, and this provided a protective effect from heparin-induced thrombocytopenia [30]. Various explanations have been postulated to explain the effect of these amino acid substitutions: introduction of torsional stress, loss of positive charge, modification of ligand binding capacity or an effect on compartmentalization of CD148 into a membrane signaling complex. It has also been suggested that ectodomain dimerization may regulate phosphatase activity, as has been reported for SAP1 and GLEPP1, which are closely-related to CD148 [31,32]. Further investigation is required to determine the precise mechanism; however, the effect of substitutions suggests that this is a key region involved in the activation/inactivation process of CD148. It is conceivable that antibody binding may interfere with these events, and this could underlie the mechanism of action of the Ab1 monoclonal antibody.

Other studies have also demonstrated biological effects with CD148-directed antibodies that are of therapeutic relevance. In studies of T cell signaling, CD148 negatively regulated T cell receptor activation and this effect was neutralized by an anti-CD148 antibody (clone A3) leading to increased T cell proliferation and increased expression of T cell surface antigens [33]. Also, expression of CD148 mRNA is upregulated in diseased joints of mice with experimental arthritis and in human arthritic joints, primarily on macrophages and T cells, where it regulates the inflammatory response, and has been proposed as a therapeutic target [34]. Treatment of macrophages with an anti-CD148 monoclonal antibody inhibited macrophage activation, specifically chemotaxis and spreading, induced by the cytokine colony stimulating factor (CSF-1), which taken together suggests that anti-CD148 antibodies may have a potential use in arthritis or other inflammatory diseases [35]. In addition to the biological effects observed with anti-CD148 antibodies, effects with cyclic peptides have also been reported; however, these effects require exceptionally high concentrations (160 μM), raising the possibility of off-target effects [36].

Studies of knock-out mice have also led to the proposition that CD148-blocking drugs may have potential as therapeutics for asthma, diabetes, and thrombosis, and biological agents targeting the ectodomain may offer advantages over small molecules. Genetic inactivation of the *PTPRJ* gene, which encodes CD148, protected mice from airway hyper-responsiveness in two different asthma models. Evidence indicated the protective effects were mediated by loss of CD148 regulation of Src family non-receptor tyrosine kinases in airway smooth muscle and a consequent reduction in contractility, rather than a dampened immune response [37]. In two other independent studies of *PTPRJ* knock-out mice on a high-fat diet, knock-out mice displayed enhanced insulin sensitivity and improved glucose tolerance, via effects on insulin signaling in skeletal muscle, liver, and adipose tissue [38,39]. In addition, a recent study reported that leptin signaling is enhanced in *PTPRJ*-deficient mice, and they exhibit lower weight gain than wild-type mice, because of a reduced food intake, mainly through effects on signaling in liver and brain [40]. Studies of thrombosis and haemostasis in CD148-deficient mice have identified that while deficient mice lacked a severe susceptibility to bleeding, thrombus formation was significantly delayed, peak thrombus size was reduced, and thrombi receded more rapidly [41,42]. Together, these exciting results suggest many potential therapeutic opportunities for CD148 blocking drugs, and targeting the ectodomain using monoclonal antibodies offers advantages in terms of achieving specificity and circumventing issues related to highly-charged small molecule phosphatase inhibitors lacking cell-permeability.

## 4. Therapeutic Potential of Biotherapeutics Targeting VE-PTP (*PTPRB*)

Vascular endothelial protein tyrosine phosphatase (VE-PTP) is an endothelial cell-specific RPTP that plays important roles in maintaining vascular integrity and angiogenesis. VE-PTP associates with vascular endothelial cadherin (VE-cadherin), a junctional adhesion molecule that is key for maintenance of vascular integrity, and also regulates both the angiopoietin receptor Tie-2 and VE growth factor receptor-2 (VEGFR-2). In recent studies, the potential of anti-VE-PTP antibodies, and a VE-PTP inhibitor, have been evaluated for use in vascular diseases and as therapeutic agents against breast cancer metastases, macular edema, neovascularization in the eye, and stroke [39,40,41,42,43].

**Figure 3:**
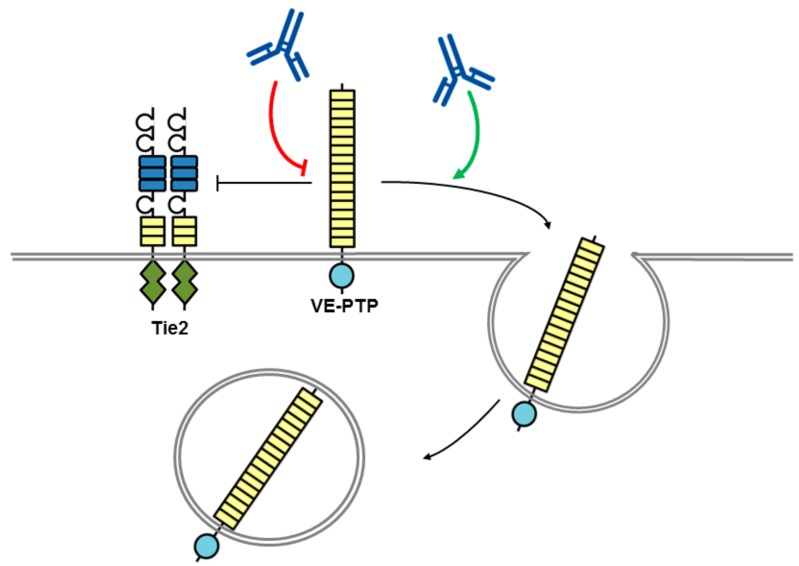
Potential mechanism of action for VE-PTP antibodies with biological effects. An antibody to the ectodomain of VE-PTP may displace VE-PTP from Tie2 triggering Tie-2 activation and VE-PTP endocytosis. Alternatively, phosphatase activity may be modulated by a VE-PTP domain directed antibody. The tyrosine kinase receptor Tie2 is shown as a dimer with a split tyrosine kinase domain (green) and an extracellular domain consisting of three epidermal growth factor (EGF)-like domains (blue rectangles) flanked by Ig1–3, followed by three fibronectin type III-like domains (yellow rectangles. RPTP domains are represented by objects as detailed in Figure 1.

Antibodies directed to the VE-PTP ectodomain trigger blood vessel enlargement in allantois explants from mouse embryos, mimicking the effects observed with deletion of the VE-PTP gene [43]. Analysis of the mechanism determined that the presence of the angiopoietin receptor tyrosine kinase Tie-2 was required, and that the antibodies selectively displace VE-PTP from Tie-2 triggering Tie-2 activation, and VE-PTP endocytosis, its down-regulation from the cell surface and subsequently degradation (Figure 3). In addition, VE-PTP blocking antibodies counteracted vascular leakage induced by inflammatory mediators and leukocyte transmigration through the endothelial cell barrier was reduced again by a mechanism involving Tie-2 [44]. The same anti-VE-PTP antibody previously shown to activate Tie-2 has been tested in mouse models of neovascular age-related macular degeneration (AMD). AMD is a leading cause of irreversible blindness characterized by abnormal growth of new blood vessels under, or within, the macula of the retina that is responsible for high-resolution vision. Intraocular injection of the antibody suppressed ocular neovascularization, and similar results were obtained with systemic administration of a small molecule VE-PTP inhibitor, AKB-9778, which also suppressed VEGF-induced vascular leakage that is relevant to diabetic macular edema [45]. The studies demonstrate that blocking VE-PTP either via an antibody or small molecule has the potential to be of therapeutic benefit clinically. In a phase I dose-escalation clinical trial with systemic administration of AKB-9778 over four weeks no safety concerns were identified [46]. A phase II trial has also been conducted assessing the efficacy of AKB-9778 alone or in combination with ranibizumab, a VEGF-neutralizing antibody, in patients with diabetic macular edema (DME) [47]. The study results indicated that monotherapy with the dose of AKB-9778 used in the study was not a viable approach to treat DME; however, the combination therapy of systemic AKB-9778 and intraocular injections of ranibizumab resulted in a significantly greater reduction in DME than ranibizumab alone. It would be of interest to establish if an anti-VE-PTP antibody delivered by intraocular injection is more effective than systemic AKB-9778, and whether such an agent could offer some advantage via its inherent high affinity and selectivity for VE-PTP.

In addition to eye disease, the AKB-9778 inhibitor has been evaluated in mouse breast cancer metastasis models and experimental models of stroke. In vitro and in vivo studies in breast cancer models showed that the drug impaired angiogenesis and slowed growth of micrometastases by limiting extravasation of tumour cells. The drug also enhanced tumour perfusion which was viewed as a benefit for enhancing responses to cytotoxic treatments [48]. In studies of experimental stroke, a disorder associated with disruption of the blood-brain barrier, increased permeability and stroke size were rescued by activation of Tie2 signaling using the VE-PTP inhibitor [49]. Therefore, both metastasis and stroke may be other therapeutic applications in which anti-VE-PTP antibodies have potential.

## 5. Therapeutic Potential of Biotherapeutics Targeting RPTPσ (*PTPRS*)

The receptor-type protein tyrosine phosphatase sigma (RPTPσ) functions in the nervous system to control axon growth and repair, and several recent studies have explored the potential of pharmacological intervention at this receptor as an approach to enhance neuronal regeneration following injury or disease. In addition, the identification of RPTPσ in joint-lining cells, called fibroblast-like synoviocytes (FLS) has led to the suggestion that targeting RPTPσ might provide a novel approach to treatment of rheumatoid arthritis; while other recent reports have provided evidence that RPTPσ is important for suppressing immune and autoimmune responses, and selective activation of this pathway might be an effective treatment for multiple sclerosis and related disorders. Here we discuss these exciting areas and progress in development of biological agents targeting RPTPσ.

**Figure 4:**
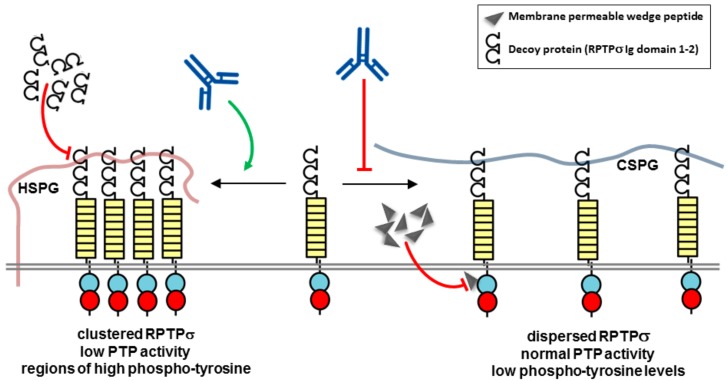
Potential mechanism of action of biotherapeutics targeting PTPRσ. Model showing heparan sulfate proteoglycans (HSPGs) acting *in cis* promoting PTPRσ oligomerization, while chondroitin sulfate proteoglycans (CSPGs) acting *in trans* oppose this effect. A decoy protein or antagonist peptide corresponding to the ectodomain may be used to block HSPG effects. A membrane permeable RPTPσ wedge domain peptide may also be used to block RPTPσ. RPTP domains are represented by objects as detailed in Figure 1.

RPTPσ is a member of the leukocyte antigen-related (LAR) subfamily of RPTPs (type IIa) along with RPTPδ and LAR. This subfamily of RPTPs has an ectodomain composed of three immunoglobulin-like (Ig) repeats and eight fibronectin type III–like (FN3) repeats. The cytoplasmic portion contains a tandem pair of PTP domains (D1 and D2), of which only the membrane proximal domain (D1) is catalytically active. RPTPσ binds the glycosaminoglycans (GAGs) side-chains of proteoglycans, including chondroitin sulfate proteoglycan (CSPG) and heparan sulfate proteoglycan (HSPG), with CSPGs typically inhibiting and HSPGs promoting axon extension (Figure 4). The GAG binding site is located in the first Ig-like domain (Ig1) and contains a cluster of conserved lysines and arginines, which are involved in an interaction with chondroitin sulfate side-chains [50]. A localization model, involving either clustering or repelling of RPTPσ, explaining the distinct functional effects of HSPGs and CSPGs on axonal growth has been proposed in which HSPGs acting in *cis* on the cell surface promote RPTPσ oligomerization, while CSPGs acting in *trans* presented by the extracellular matrix oppose this effect [51]. The different effects are explained by the highly sulphated GAG side-chains in HSPGs, but not in CSPGs, leading to RPTPσ clustering and consequently uneven distribution of phosphatase activity over the cell surface. This would create regions of higher phosphotyrosine levels, where RPTPσ has been depleted, which could enhance signaling events involved in neuronal extension (Figure 4). Early studies of neurons from RPTPσ knock-out mice determined that RPTPσ gene disruption enhances the ability of axons to penetrate regions of neural lesions enriched in inhibitory CSPGs, and recognised that function blocking antibodies, soluble ectodomain constructs or small molecule compounds capable of blocking CSPG effects would provide a new therapeutic approach to neural regeneration [50,52] (Figure 4). Several recent studies have put this principle into practice. In an adult rat spinal cord injury model, a membrane permeable peptide to the RPTPσ wedge domain restored substantial innervation of the spinal cord below the level of the injury and facilitated functional recovery of both locomotor and urinary systems [53]. Similarly, in the mouse heart, following surgery to induce a myocardial infarction, the permeable RPTPσ wedge domain peptide restored sympathetic innervation mimicking the effects of genetic deletion of RPTPσ. The re-innervation also rendered hearts resistant to isoprenaline-induced arrhythmias [54]. Although the precise mechanism by which the RPTPσ wedge domain peptide functions is unknown, and specificity may be an issue since several RPTPs have a putative wedge domain [55], the results highlight the therapeutic potential of the approach in patients who have suffered a spinal cord injury or myocardial infarction.

Function-regulating antibodies targeting the RPTPσ ectodomain offer a potential alternative therapeutic approach. Wu et al. developed a split luciferase assay to monitor RPTPσ dimerization in living cells with the objective of using the system to identify RPTPσ antibodies that modulate its activity [56]. They demonstrated that heparin, an analog of heparan sulfate, promoted RPTPσ dimerization whereas chondroitin sulfate inhibited dimerization and increased RPTPσ phosphatase activity. Several antibodies were tested and an antibody (4.5H5) was identified that induced dimerization and promoted neurite outgrowth in neuroblastoma SH-SY5Y cells, consistent with therapeutic potential [49].

Recently, with the identification of high RPTPσ expression levels in arthritic fibroblast-like synoviocytes (FLS), specialized synovial lining cells that in rheumatoid arthritis have a major role in destructive joint inflammation, RPTPσ has been identified as a therapeutic target for rheumatoid arthritis [57]. As in neurons, RPTPσ activity is regulated by proteoglycans. The large CSPG aggrecan is the main proteoglycan in cartilage [58], while other cell surface proteoglycans, such as the syndecan family of molecules, mainly contain heparan sulfate side-chains and are involved in connecting the surface of cells to the underlying extracellular matrix, together with a wide range of other biological functions. In a mouse model of rheumatoid arthritis syndecan-4 has been identified as a prominent molecule in fibroblast attachment and cartilage damage, a step which is recognised as irreversible and is a ‘point of no return’ in joint destruction in arthritis [59]. Doody and colleagues have elucidated the mechanisms involved, identifying a key role for RPTPσ and have demonstrated that an RPTPσ decoy protein reduces arthritis severity (Figure 4). Antisense knock-down experiments of syndecans on FLS determined that syndecan-4 is the HSPG that physiologically regulates RPTPσ in FLS. Further experiments demonstrated in vivo that the decoy protein reduces FLS invasion of cartilage and decreases arthritis severity in a mouse model of arthritis [57]. Together these exciting findings indicate that targeting the RPTPσ syndecan-4 interaction in FLS could be an effective treatment for rheumatoid arthritis. It has been proposed that suitable agents could be combined with existing disease-modifying anti-rheumatic drugs, or used as a monotherapy in patients where the disease is driven more by FLS activation, and the novel approach would have the advantage of not causing significant immune suppression. However, as we discuss below, such an approach may not be devoid of immunological modulatory effects as it has been reported recently that RPTPσ is an important inhibitory receptor on several types of immune cells and has pro-inflammatory activities of certain cell types.

While blocking the constitutive interaction of RPTPσ and syndecan-4 has been demonstrated as a potential therapeutic approach for rheumatoid arthritis, recent studies have uncovered an important role of RPTPσ in several immune cell types, leading to the proposition that targeting RPTPσ may also be a therapeutic strategy for some inflammatory or autoimmune disorders. Bunin et al. demonstrated that RPTPσ is expressed on plasmacytoid dendritic cells (pDC), which are key producers of interferon α (IFNα) in response to viruses [60]. Activation of the pDC led to a rapid loss of RPTPσ cell surface expression either through internalization or shedding from the membrane, and loss of expression correlated with hyper-responsiveness of pDCs, and IFNα production. The authors also found that RPTPσ deletion from dendritic cells in mice, also deficient in the related LAR phosphatase, was associated with mild colitis, which correlates with a previous study reporting that RPTPσ knockout mice develop mild colitis and that polymorphisms in the *PTPRS* gene are linked to ulcerative colitis [61]. As with the studies of RPTPσ regulation in neurons and fibroblasts discussed above, the HSPG ligand, glypican, and CSPG ligand, neurocan, activated and inhibited RPTPσ signaling, respectively. An antibody to RPTPσ elicited effects consistent with an RPTPσ agonist, inhibiting pDC activation, leading to the suggestion that this could be a therapeutic approach for colitis (Figure 4). The authors also suggested that the opposite strategy of RPTPσ selective inhibition might be used to boost the immune response for treatment of chronic viral infection or tumours. In addition, Ohtake et al. have identified that RPTPσ affects activation of dendritic cells, T lymphocyte differentiation and activity of regulatory T cells [62]. Deletion of RPTPσ in transgenic mice or use of a 17 amino acid antagonist peptide (termed sIg1; KPRVTWNKKGKKVNSQR), corresponding to the first Ig domain of RPTPs, that is essential for ligand binding, induces a pro-inflammatory state that exacerbates symptoms of experimental autoimmune encephalomyelitis (EAE), an animal model of multiple sclerosis (MS). The authors highlight that an agent with the opposite biological effects, namely selective activation of the RPTPσ pathway, may become an effective strategy for MS.

## 6. Therapeutic Potential of Biotherapeutics Targeting CD45 (*PTPRC*)

**Figure 5:**
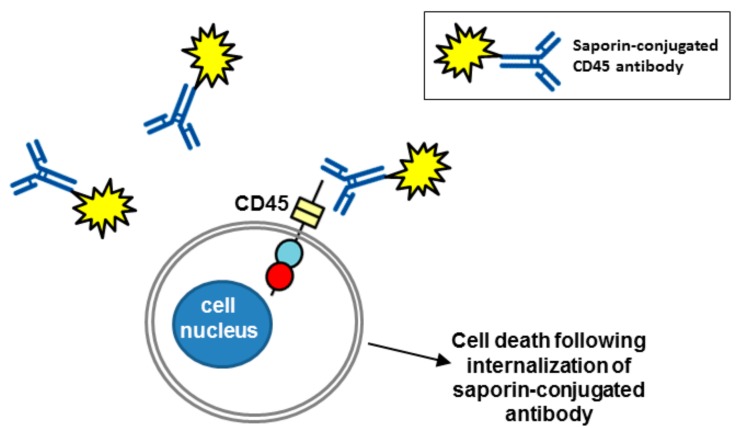
A CD45 antibody conjugated with saporin as a cell-specific immunotoxin. Saporin conjugated to a CD45 antibody gets rapidly internalized via endocytosis (not shown) leading to cell death. RPTP domains are represented by objects as detailed in Figure 1.

The RPTP CD45 (also called the leukocyte common antigen) is expressed on all haematopoietic cells, with the exception of platelets and erythrocytes, and functions as a key regulator of T and B cell signalling. It is the sole member of the R1/R6 subtype of RPTPs and consists of an extracellular region, short transmembrane segment and tandem PTP domains in the cytoplasmic region. Multiple isoforms of CD45 are generated by complex alternative splicing of exons in the extracellular domain of the molecule, which are expressed in a cell type specific manner depending on the cell differentiation and activation status [63]. It has been the target of immunotherapeutics in several studies as a conditioning pre-treatment prior to haematopoietic stem cell transplantation (HSCT), which is used for malignant and non-malignant haematological disorders. The purpose of conditioning is to destroy haematopoietic stem cells in the host’s bone marrow and facilitate engraftment through host immunosuppression. Current approaches to conditioning involve total body irradiation with or without chemotherapy drugs and since these are non-targeted they can have serious adverse effects through cytotoxic and genotoxic effect on healthy tissue. Newer antibody-based conditioning agents are expected to have much less off-target toxicity and anti-CD45 monoclonal antibodies have received much attention in this role. Unlabelled anti-CD45 antibodies have been tested; however, they depleted only lymphoid cells and additional chemotherapy was required to deplete haematopoietic stem cells [64,65]. Anti-CD45 antibodies radiolabelled with ^131^I have been tested in phase I and II trials with chemotherapy agents as conditioning agents before HSCT in acute leukaemia [66,67]. More recent efforts have focussed on antibodies labelled with α-emitters, rather than β-emitters, since they have a shorter range and higher linear energy transfer meaning that there is a reduction in bystander toxicity where non target cells close to target cells are affected [68]. Recently, Palchaudhuri et al. reported exciting results using a CD45 antibody conjugated with the plant toxin saporin (CD45-SAP) as a haematopoietic-cell-specific immunotoxin [69] (Figure 5). Saporin produces its cytotoxic effects by both inhibiting protein synthesis via its N-glycosidase activity, which cleaves the 28 S rRNA of eukaryotic ribosomes, and by genomic DNA fragmentation via DNAase activity. Alone it is not internalised efficiently and is non-toxic, but on conjugation to an antibody to a cell surface antigen that gets rapidly internalized via endocytosis it becomes a potent toxin [70,71]. The CD45-SAP agent efficiently conditions immuno-competent mice for HSCT and minimizes undesirable toxicity compared with conventional total body irradiation conditioning, indicating this may be a promising approach in the future.

## 7. Therapeutic Potential of Biotherapeutics Targeting RPTPγ and RPTPζ (*PTPRG* and *PTPRZ1*)

The RPTPs RPTPγ and RPTPζ form the R5 RPTP subfamily, with an extracellular carbonic anhydrase domain, a fibronectin type III like-domain and an intracellular tandem phosphatase domain. Both molecules are highly expressed in the central nervous system (CNS), and RPTPγ is expressed widely in many peripheral tissues, including leukocytes, epithelial cells, and endocrine cells of various organs [72,73,74]. RPTPγ is known to act as tumour suppressor in various cancers [75], and a recent report has demonstrated that RPTPγ-directed monoclonal antibodies have a potential use as a tool for biomarker detection in chronic myeloid leukaemia (CML) [76]. In a group of newly diagnosed CML patients, Vezzalini et al. used an RPTPγ antibody (TPγ B9-2) to confirm down-regulation of RPTPγ at diagnosis and demonstrated that following tyrosine kinase inhibitor treatment its expression recovered, together with a return to normal haematopoiesis [76].

**Figure 6:**
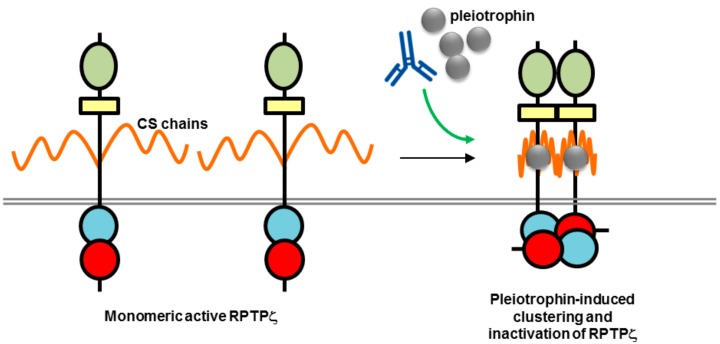
Potential mechanism of action of biotherapeutics targeting RPTPζ (adapted from reference [68]). The high chondroitin sulfate (CS) modification of RPTPζ is thought to maintain the RPTP in a monomeric active form by electrostatic repulsion. Binding of a ligand such as pleiotrophin is thought to neutralize the negative charge and induce clustering and inactivation of phosphatase activity. An antibody may be used to mimic or enhance ligand-induced clustering. RPTP domains are represented by objects as detailed in Figure 1.

While both RPTPγ and RPTPζ have high-level CNS expression in common, they differ in their expression patterns, with RPTPγ found almost exclusively on neurons and RPTPζ localized on both glial cells, specifically oligodendrocytes and their precursor cells that are important in myelination of nerve axons, and neurons [77,78,79]. Another key difference is that RPTPζ, unlike RPTPγ, is heavily modified with chondroitin sulfate side-chains, which are required for high-affinity binding of inhibitory ligands, such as pleiotrophin, midkine and interleukin-34 [79]. A recent study by Kuboyama et al. presented an elegant model of RPTPζ regulation by chondroitin sulfate and pleiotrophin and its relevance to demyelination in brain regions in multiple sclerosis (Figure 6). The chondroitin sulfate modification of RPTPζ was found to be essential for maintaining RPTPζ in a monomeric active state associated with inhibition of oligodendrocyte precursor cell (OPC) differentiation. Pleiotrophin was found to be the ligand responsible for inducing OPC differentiation, and associated myelination, during brain development, by binding to the negatively charged chondroitin sulfate side-chains, and inducing clustering and inactivation of RPTPζ by the head-to-toe model [10]. In MS it is thought that chondroitin sulfate proteoglycans in lesions interfere with the binding of pleiotrophin to RPTPζ causing an inhibitory effect on remyelination [80]. An antibody to RPTPζ tested in this study did not enhance OPC differentiation; however, it is possible in the future that modulation of this pathway may be achieved with other biotherapeutics, monoclonal antibodies, or fusion proteins, as a strategy for tackling MS.

## 8. Conclusions

A wide range of therapeutic opportunities for biotherapeutics targeting RPTPs have been reported in a broad range of disease conditions in the scientific literature, including: asthma, diabetes, thrombosis, diabetic eye disease, cancer metastasis, stroke, rheumatoid arthritis, multiple sclerosis, spinal cord injury, myocardial infarction, and as conditioning agents prior to HSCT. RPTP ectodomains are obvious targets, and the biotherapeutic approach circumvents the issues associated with getting small-molecule phosphatase inhibitors across the plasma membrane and achieving specificity. Monoclonal antibodies, decoy fusion proteins, and peptides have all been used to modulate RPTP ligand binding and regulation of catalytic activity to achieve therapeutically relevant effects. The heterogeneity of RPTP ectodomains and different isoforms of some RPTPs may also provide an additional degree of specificity that can be exploited in the future. With the exciting recent developments in the field of biotherapeutics targeting RPTPs and development of small-molecule PTP inhibitors [16,17], the race is on for the first approved PTP-targeted drug.
